# Pathophysiology and Molecular Signalling in Osteoporosis: Linking Risk Factors to Bone Loss

**DOI:** 10.1111/jcmm.71155

**Published:** 2026-04-26

**Authors:** Pramoda G., Kamini Shivhare, Piyush Kumar Gupta, Amirhossein Sahebkar, Prashant Kesharwani, Rahul Shukla

**Affiliations:** ^1^ Department of Pharmaceutics National Institute of Pharmaceutical Education and Research (NIPER)‐Raebareli Lucknow Uttar Pradesh India; ^2^ Department of Biotechnology Motilal Nehru National Institute of Technology Allahabad Prayagraj Uttar Pradesh India; ^3^ Biotechnology Research Center, Pharmaceutical Technology Institute Mashhad University of Medical Sciences Mashhad Iran; ^4^ Next‐Generation Translational Nanomedicine Laboratory, Department of Pharmaceutical Sciences Dr. Harisingh Gour Vishwavidyalaya (A Central University) Sagar Madhya Pradesh India; ^5^ Department of Research and Development Asia University Taichung Taiwan

**Keywords:** bone metabolism, bone remodelling, osteoporosis, pathophysiology, RANKL–OPG axis, risk factors, vitamin D, Wnt/β‐catenin signalling

## Abstract

Osteoporosis is a prevalent skeletal disorder characterised by progressive reduction in bone mass, microarchitectural deterioration, and increased fracture susceptibility. In India, approximately one‐third of the elderly population is affected by bone‐related disorders, and the global burden of osteoporosis continues to rise. Despite its significant impact on morbidity, mortality, and quality of life, current diagnostic approaches and therapeutic strategies remain suboptimal due to limitations such as prolonged treatment duration, poor patient adherence, low oral bioavailability of first‐line therapies, and potential cardiovascular risks associated with some anti‐resorptive agents. Given these challenges, there is an urgent need to develop safer and more effective preventive and therapeutic interventions. However, rational drug design and targeted therapies require a comprehensive understanding of osteoporosis pathophysiology. In this review, we provide an integrated overview of normal bone remodelling, key cellular players, and the mechanisms underlying impaired bone homeostasis in osteoporosis. We discuss the roles of osteoblasts, osteoclasts, and their progenitor cells, along with critical regulatory factors governing their differentiation and function. Later, we have discussed the core mechanisms and pathways involved in the pathophysiology of osteoporosis, including the RANKL–OPG axis, Wnt/β‐catenin signalling, Parathyroid hormone (PTH)/PTH1R (cAMP/PKA vs. sustained Ca^2+^/PKC) signalling, TGF‐β/BMP–SMAD signalling, etc. Furthermore, we summarise the major risk factors, including ageing, sex hormones, nutritional deficiencies, lifestyle factors, and comorbidities and delineate their mechanistic links to bone loss. This comprehensive synthesis aims to enhance understanding of osteoporosis pathogenesis and to facilitate the identification of novel molecular targets for improved therapeutic strategies.

**Abbreviations**
5α‐DHT: 5α‐dihydrotestosterone
AMPK: AMP‐activated protein kinase
ANGPT: Angiopoietins
AR: Androgen receptor
BLC: Bone lining cells
BMD: Bone mineral density
BMP: bone morphogenic proteins
BMU: Bone remodelling Unit
BSP: Bone sialoprotein
CD: Celiac disease
DKK1: Dickkopf‐1
Dll: Delta‐like
Dvl: Dishevelled
Emcn: Endomucin
ER‐α: Oestrogen receptor‐α
FAK: Focal adhesion kinase
GC: glucocorticoid
GH: Growth hormone
GR: Glucocorticoid receptors
HIF: Hypoxia‐inducible factor
HSC: Haematopoietic stem cells
IGF‐1: Insuline growth factor‐1
Ihh: Indian Hedgehog
IL‐1: interleukin‐1
IL‐6: interleukin‐6
ILK: Integrin‐linked linase
LRP 5/6: Low‐density lipoprotein receptor‐related proteins 5/6
M‐CSF: Macrophage colony‐stimulating factor
MSC: Mesenchymal stem cells
OPG: Osteoprotegerin
PDGF: Platelet‐derived growth factor
PIGF: Placental growth factor
PKA: Protein kinase A
PKC: Protein kinase C
PTH: Parathyroid hormone
PTHrP: parathyroid hormone‐related protein
RANKL: Receptor activation of NF‐кB ligand
ROS: Reactive oxygen species
SMAD: Suppressor of Mothers against decapentplegic
SOST: sclerostin
TGF‐b: Transforming growth factor‐β
TNF‐α: Tumour necrosis factor‐α
TSH: Thyroid stimulating hormone
VDR: Vitamin D receptor
VEGF: Vasluar endothelila growth factor

## 1 Introduction

Bone is a vital structural component of the body, having the primary role in protecting organs, regulating ions and forming blood cells [1, 2]. The bone is a dynamic system that undergoes continuous remodelling, and osteoblasts and osteoclasts are the major bone cells involved in it [3]. It was estimated that the global rise in the acute or long‐term symptoms of a bone‐related fracture was up to 70% since 1990 [4]. Around 37 million fractures are reported annually, with 1 in 3 women and 1 in 5 men aged over 50. According to the International Osteoporosis Foundation, 6.3% of men and 21.2% of women over the age of 50 are affected by osteoporosis globally. It was also predicted that the hip fracture case may rise to 7.3–21.3 million by 2050. The lifetime risk of hip fracture in white women is 1 in 6 when compared to 1 in 9 risk of breast cancer diagnosis [5, 6]. In the Indian elderly population, 49.9% individuals are suffering from bone‐related issues, and around 18% of the population is affected by osteoporosis. The prevalence of osteoporosis in postmenopausal women is 33.1% and in women aged above 60, the incidence increased to 37.0% [7]. These figures indicate the need for attention for bone health in India and across the world. Among the bone‐related diseases, Osteoporosis is a condition where the bone density is reduced, with increased susceptibility to fracture and affects the quality of life. The condition remains asymptomatic until the disease condition worsens and a fracture occurs, which makes early diagnosis and intervention challenging. The major issues of the disease, like expensive and complex diagnostic techniques, limited therapeutic options, the extended treatment period, and slow/delayed recovery, need to be addressed [8]. Among them, therapy standards are a major challenge that requires the development of better therapies that overcome the existing issues, including the demand for prolonged therapy combined with low adherence, poor and inconsistent bioavailability of drugs of first line therapy, and associated risks of gastrointestinal disturbance, osteonecrosis of the jaw, cardiovascular complications like stroke and coronary events, and a potential increase in cancer risk [5]. However, prior to the development of better therapy, it is necessary to have a detailed understanding of pathology and pathways, which act as targeted factors in the regulation and management of osteoporosis. Considering the need, we have summarised the major pathways and factors that have a pivotal role in the development of osteoporosis. The bone integrity was primarily maintained by the bone‐forming cells (osteoblasts) and the bone‐resorption cells (osteoclasts). The impairment in the activity of these cells will be a possible cause of the bone with degraded architecture. So, we have discussed the various factors that influence the differentiation, proliferation, and cell function of the osteoclasts and osteoblasts. Apart from them, certain risk factors like age, sex, hormones, diet, lifestyle, etc., can cause osteoporosis [9, 10]. In this current review, we have discussed the process of bone formation and deterioration, the role of osteoclasts and osteoblasts in maintaining the integrity of bone architecture. Then we have discussed in detail various risk factors and their mechanism behind the development of osteoporosis. Later, we have given a complete discussion on the major pathways like Wnt/β‐catenin signalling, TGF‐β/BMP–SMAD signalling, Oestrogen/Androgen signalling, etc., involved in the differentiation and cell function of the bone cells and osteoporosis development.

## 2 Bone Cells and the Mechanism of Bone Formation

The bone is the strong and rigid connective tissue of the body made up of inorganic and organic components. The strength and rigidity are provided by inorganic components, and organic components provide flexibility to the bone structure. The extracellular bone matrix comprises 60% of inorganic substances, 25% of organic substances, and 5% of water in terms of bone weight. The inorganic components of the bone matrix are crystals of hydroxyapatite (calcium and phosphate), magnesium carbonate, sodium, and potassium ions, whereas organic components include collagen and non‐collagen substances. The non‐collagenous proteins of the bone matrix are osteonectin, osteocalcin, and sialoprotein; and 90% of the collagenous substance is type‐I collagen, which is produced by osteoblasts [11, 12]. The process of formation of bone is called ossification, and the primary stage of bone development occurs through two different processes called intramembranous ossification and endochondral ossification. The intramembranous ossification is responsible for the formation of the skull, cranial vault, clavicle and facial bone, which involve osteoblasts and transcription factor Runx2. Endochondral ossification can be observed throughout the body, and the process involved in the development of bones like the femur, tibia, humerus, radius, etc. [13].

**FIGURE 1:**
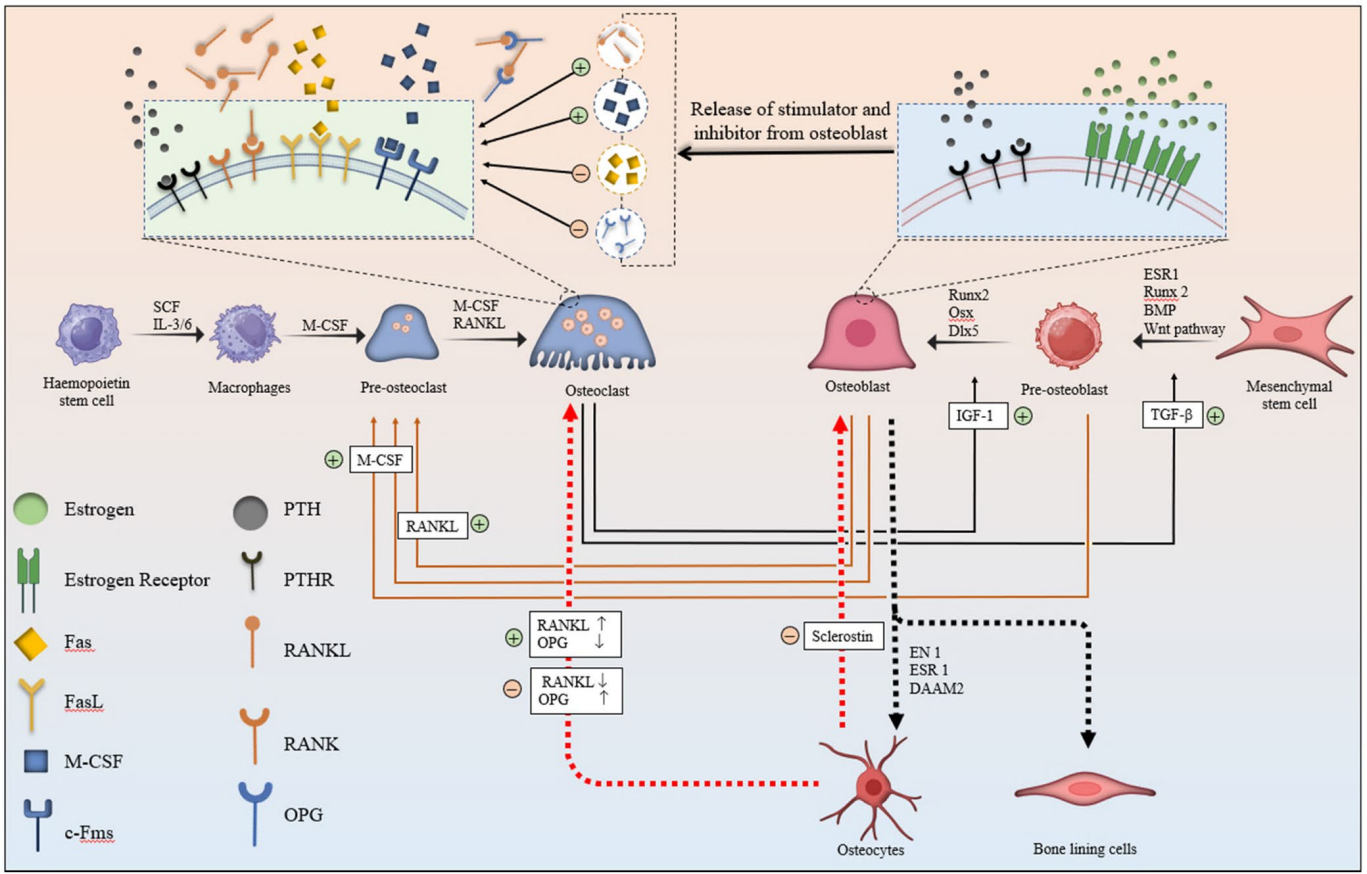
Factors that are involved in the differentiation of osteoblasts and osteoclasts cells.

**FIGURE 2:**
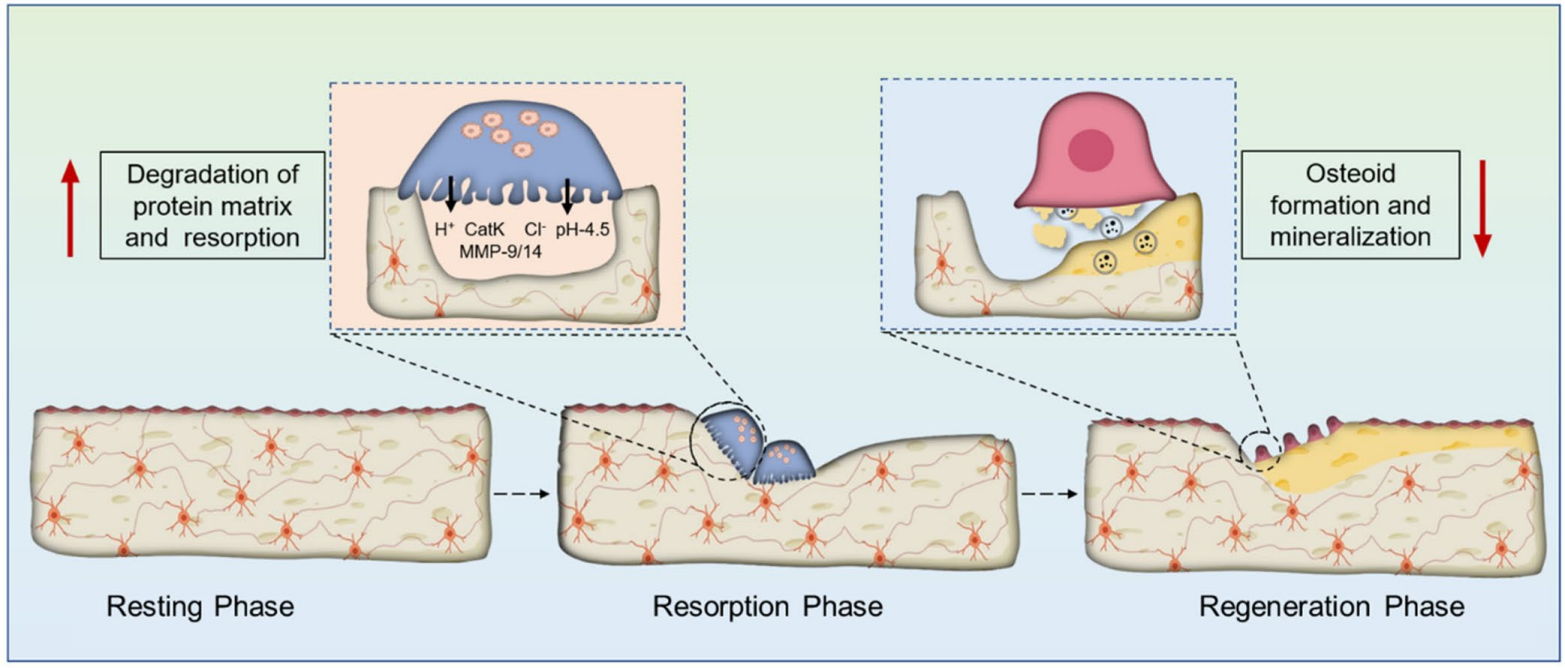
Pathophysiology of osteoporosis.

The cells involved in bone formation and remodelling are osteoblasts, osteocytes, osteoclasts, and bone lining cells [14]. The parent cells, differentiation and proliferation of bone cells, specifically osteoblasts and osteoclasts, will differ from each other and are represented in Figure 1. Osteoblasts are the bone‐forming cells derived from the mesenchymal stem cells (MSC). The MSC differentiated into osteoprogenitor cells and further matured as osteoblasts with the assistance of bone morphogenic proteins (BMPs), Runx2, Dlx5 and osterix. The mature osteoblast is responsible for bone formation, and it takes place in two phases. The initial phase is the secretion of proteins like type‐I collagen, osteonectin, osteopontin, bone sialoprotein (BSP) II, osteocalcin, decorin and biglycan, which are further deposited to form the protein matrix (Figure 2). Later, in the second phase, mineralisation of the protein matrix occurs to form bone. The mineralisation process is aided by special vesicles called matrix vesicles released by osteoblasts. It occurs in two steps: the first step is the release of calcium‐containing vesicles, the binding of vesicles to the protein matrix, and immobilisation of the calcium ions. In the second step, the phosphate ions move into the vesicles, and the supersaturation of the calcium and phosphate is achieved inside the vesicles. The supersaturation leads to the formation of the hydroxyapatite crystals, which rupture the vesicular structure and release into the protein matrix to achieve the mineralisation process and complete the bone formation processes [15, 16]. The cells involved in bone resorption are called osteoclasts, and they are large multinucleated cells formed in the bone remodelling compartment from the precursor cells called haematopoietic stem cells (HSC). Along with HSC, macrophage colony‐stimulating factor (M‐CSF) and receptor activation of NF‐кB ligand (RANKL) are two essential factors for the formation of osteoclast cells. Upon maturation, they get polarised on the bone surface to form the sealing zone and ruffled surface with the assistance of actin‐rich podosomes. Later, they create the extracellular acidic microenvironment on the bone surface resulting in the chemical reaction and liberation of the calcium ion from the hydroxyapatite crystals. The osteoclast also secretes a lysosomal proteolytic enzyme called cathepsin K, which is responsible for the proteolytic cleavage of the organic bone matrix. The overall process causes demineralisation and degradation of the bone matrix called bone resorption [17, 18, 19].

The osteocytes and bone lining cells (BLC) are bone cells that preserve the bone structure and integrity. They are entrapped in the bone matrix (lacunae) and they communicate signals throughout the bone via interconnected canals called canaliculi. They work as a mechanosensory component of the bone by converting the mechanical stress into biochemical signals to induce osteoblast/osteocyte‐mediated bone formation. Another post‐proliferative osteoblast cell form was the bone lining cells. They have flat and elongated structures, lining the surface of the bone when it is static. The only responsibility of the BLC is to prevent the direct interaction of the osteoclast with the bone surface and to protect the bone from resorption [11, 15, 20].

Apart from the bone cells and bone matrix, angiogenesis and vascularisation have a significant role in bone health. Bone receives around 10%–15% of the cardiac output, which supplies the nutrients, minerals and hormones required for bone formation and homeostasis, blood cell formation and maturation, and metabolic waste removal and fracture healing [21, 22]. Generally, the angiogenesis induced by the growth factor called VEGF is stimulated by the hypoxia condition. When the cellular environment is short of oxygen, the hypoxia condition will be created, which results in the release of hypoxia‐inducible factor‐1α (HIF‐1α), and later it is responsible for the activation of various proangiogenic and angiogenic genes and receptors, like vascular endothelial growth factor (VEGF), Placental growth factor (PlGF), Platelet‐derived growth factor (PDGF) and angiopoietins (ANGPT1 and ANGPT2) [23]. However, this is the common physiology observed throughout the body, but in the bone, there is a unique form of blood vessels formed called type‐H vessels. The endothelial cells of these vessels are highly expressed with endomucin and CD31, responsible for better osteogenic cell differentiation and proliferation [24].

## 3 Micro‐Pathophysiology of Osteoporosis (Cellular & Signalling Pathways)

**FIGURE 3:**
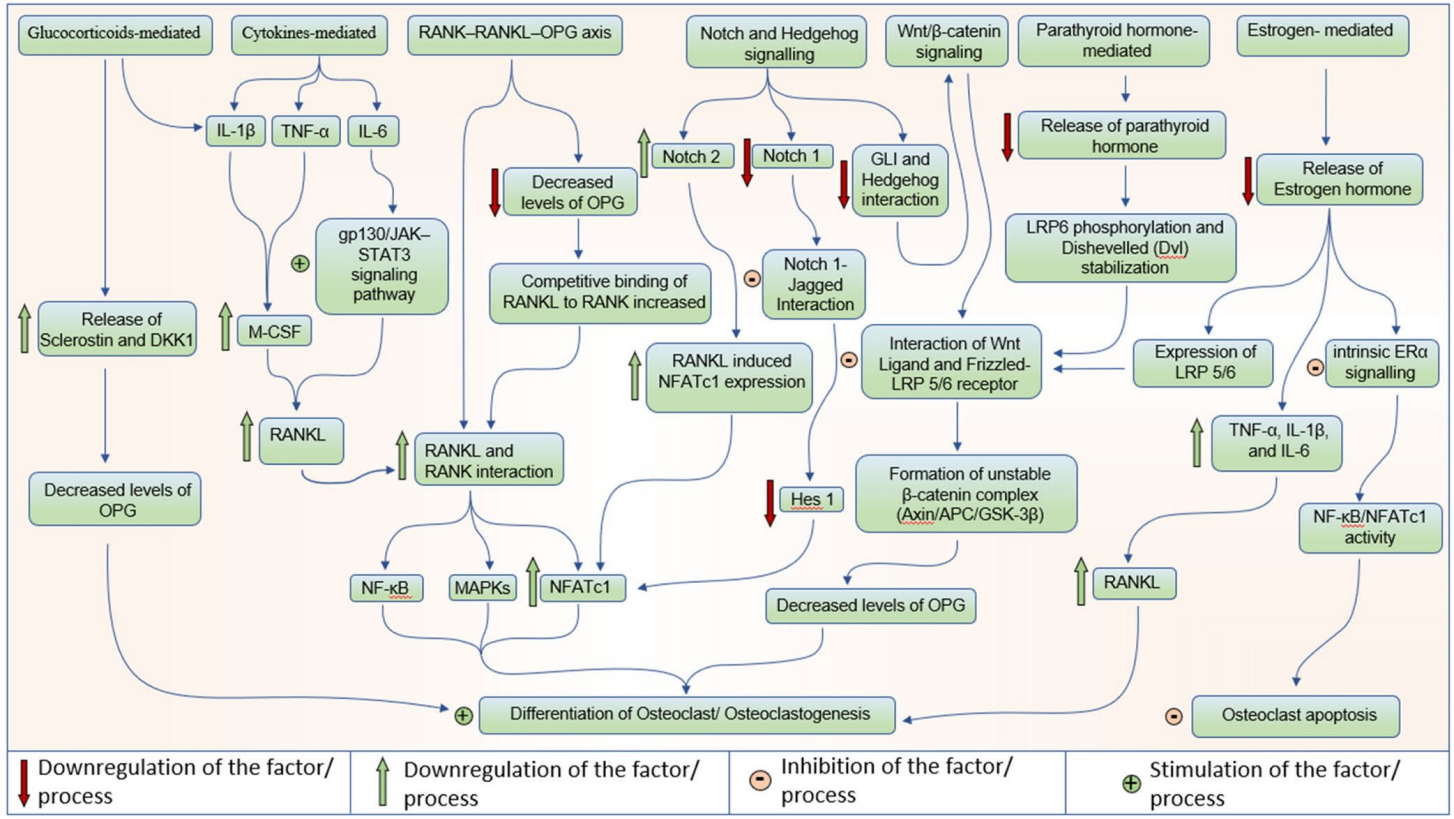
Pathways involved in the survival and function of osteoclasts.

**FIGURE 4:**
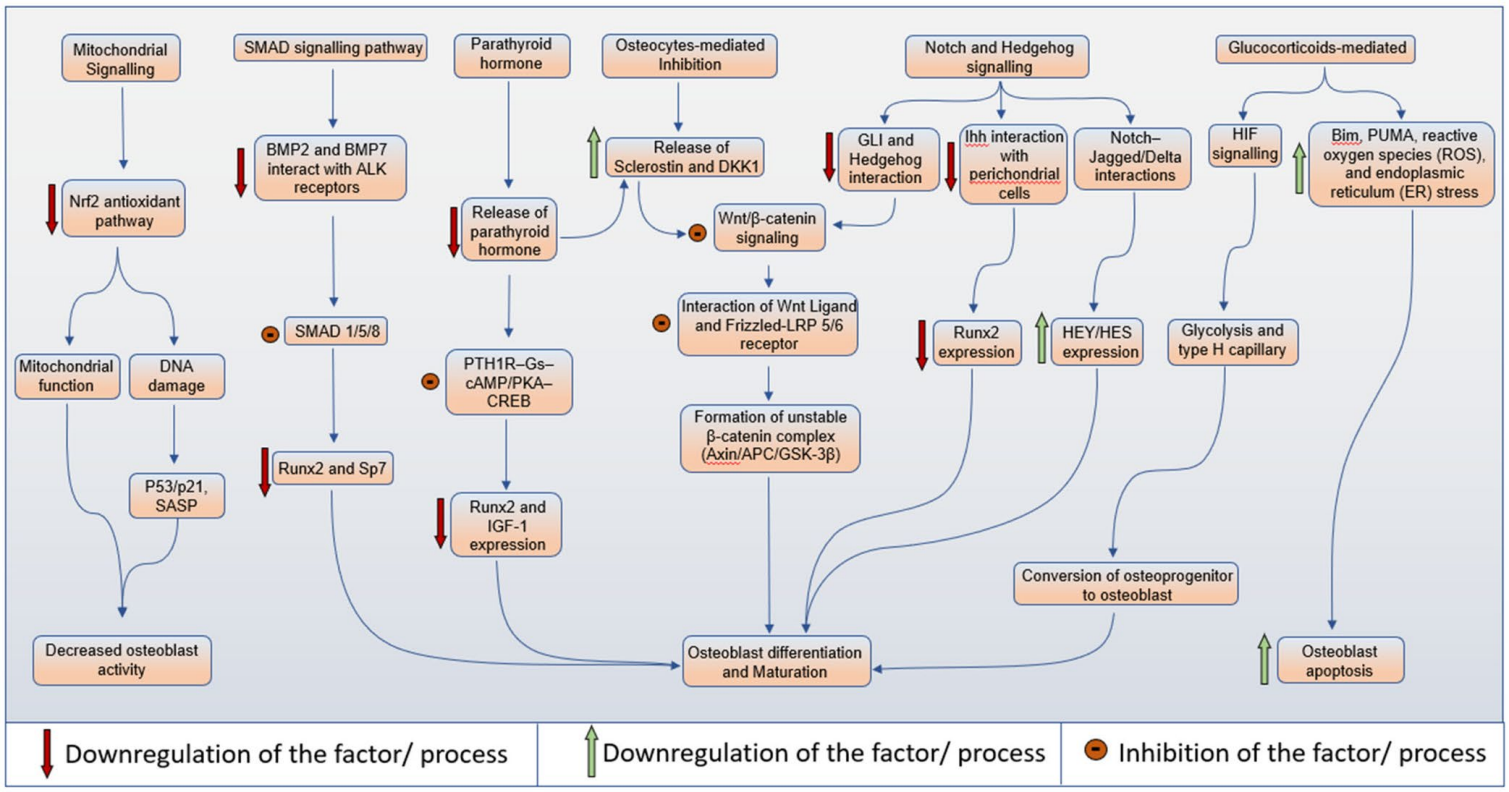
Pathways involved in the survival and function of osteoblasts.

Osteoporosis develops when there is an imbalance in the bone remodelling process. This imbalance ultimately leads to a loss of bone density [25]. A key player in this process is the RANK–RANKL–OPG signalling pathway. Here, RANKL activates NF‐κB/NFATc1, which drives the formation of osteoclasts. On top of that, when oestrogen levels drop or inflammation kicks in, the expression of OPG decreases, which only adds to the problem by promoting bone resorption (Figure 3) [26]. In contrast, the Wnt/β‐catenin pathway plays a crucial role in promoting the commitment of osteoblast lineage and the production of OPG. However, in osteoporosis, the activity of these pathways will be hindered by increased levels of antagonists like sclerostin and DKK1 (Figure 4) [27]. Hormonal signals such as PTH, oestrogen and glucocorticoids have a dual impact on bone health: while intermittent PTH and oestrogen support bone formation, a loss of oestrogen or prolonged glucocorticoid signalling can lead to increased RANKL, oxidative stress, and the death of osteoblasts B (Tables 1 and 2) [28]. Moreover, various factors including oxidative stress, inflammatory cytokines, the uncoupling of hypoxia and angiogenesis, impaired mechanotransduction, gut microbiota imbalances, and changes in epigenetics/miRNA work together to integrate both local and systemic signals that drive the complex micro‐pathophysiology of osteoporosis [29].

**TABLE 1:** Key signalling pathways in osteoporosis pathophysiology.

Pathway	Role in bone remodelling	Effect in osteoporosis	Potential therapeutic target	References
RANK–RANKL–OPG	Regulates osteoclastogenesis	↑ RANKL/↓ OPG → excessive resorption	Denosumab (anti‐RANKL)	[30]
Wnt/β‐catenin	Promotes osteoblastogenesis & OPG production	Suppressed by ↑ sclerostin/DKK1	Romosozumab (anti‐sclerostin)	[31, 32]
PTH/PTH1R	Intermittent → anabolic, sustained → catabolic	Hyperparathyroidism → resorption	Teriparatide, Abaloparatide	[33]
TGF‐β/BMP–SMAD	Regulates progenitor recruitment/differentiation	Dysregulated gradients impair remodelling	BMP‐based therapy (experimental)	[34, 35]
Glucocorticoid receptor	Modulates osteoblast/osteocyte survival	Chronic exposure → apoptosis, Wnt inhibition	GC‐sparing therapy	[36, 37]

**TABLE 2:** Emerging mechanisms in osteoporosis (micro‐pathophysiology).

Mechanism	Molecular players	Outcome
Oxidative stress	ROS, FOXO, Nrf2, p53/p21	↓ osteoblastogenesis, ↑ osteoclast activity
Inflammation	TNF‐α, IL‐1β, IL‐6, NF‐κB, JAK/STAT3	↑ resorption, ↓ bone formation
Hypoxia/angiogenesis	HIF‐1α, VEGF, type‐H vessels	Reduced osteoprogenitor support
Gut–bone axis	SCFAs, GPR41/43, microbiota composition	Modulates immune signalling & calcium absorption
Epigenetic regulation	DNA methylation, HDACs, miRNAs	Alters Runx2, Wnt, RANKL/OPG expression
Circadian rhythm	BMAL1, β2‐adrenergic signalling	Disrupted remodelling balance

### 3.1 Bone Remodelling Unit: Coupling Overview

Bone remodelling is a highly coordinated, dynamic process regulated by an interconnected network of hormonal, mechanical and metabolic signals within the bone remodelling unit (BMU), rather than isolated pathways. In osteoporosis, this equilibrium is lost as systemic risk factors converge on key signalling hubs, driving uncoupled remodelling and progressive bone loss [38, 39, 40]. Several highly conserved signalling pathways, including the RANKL–RANK–OPG axis, Wnt/β‐catenin signalling, parathyroid hormone (PTH)/PTH1R signalling, and TGF‐β/BMP–SMAD pathways, are at the centre of this regulation network. Instead of operating separately, these routes combine to produce an integrated signalling circuitry that includes feedback loops, context‐dependent modulation, and a great deal of crosstalk. Like, RANKL‐mediated osteoclastogenesis is indirectly suppressed by Wnt/β‐catenin signalling, which simultaneously increases osteoprotegerin (OPG) expression and promotes osteoblast differentiation [41, 42]. On the other hand, inflammatory mediators like TNF‐α and IL‐6 can change the balance in favour of bone resorption by inhibiting Wnt signalling while increasing RANKL expression [43]. Crucially, these core signalling hubs are disrupted by upstream modulators such as ageing, oestrogen shortage, oxidative stress, and chronic inflammation [44]. Reduced osteoblastogenesis results from the increase of reactive oxygen species (ROS) associated with ageing, which diverts β‐catenin from TCF‐mediated transcription towards FOXO‐dependent stress responses [45, 46]. In a similar vein, oestrogen insufficiency promotes osteoclast differentiation and survival by increasing RANKL expression, decreasing OPG levels, and enhancing NF‐κB activation [47, 48]. These findings demonstrate that osteoporosis is a systems‐level ailment marked by network dysregulation rather than just a disease of specific pathways. Recent advancements underscore that bone remodelling is governed by complex, intersecting regulatory layers, including osteoimmunological signalling, epigenetic modifications, and metabolic pathways like PI3K–Akt–mTOR and AMPK, which integrate environmental and intracellular signals. For instance, pro‐inflammatory cytokines enhance osteoclastogenesis through the NF‐κB/NFATc1 pathway while simultaneously inhibiting osteoblast differentiation by suppressing Runx2 and Wnt signalling. Furthermore, epigenetic regulators, such as histone modifications and microRNAs, add an additional layer of complexity by fine‐tuning the expression of essential bone remodelling genes [49, 50]. Targeted bone therapies (denosumab, romosozumab and teriparatide) succeed clinically but have inherent limitations, necessitating a tailored, systems‐level approach to signalling interactions [51, 52]. Collectively, several risk factors come together on common molecular pathways; osteoblast function is compromised, osteoclast activity is increased, and bone remodelling is compromised. As a whole, osteoporosis should be understood as a condition of integrated signalling network failure. The next sections that analyse specific pathways in relation to their interactions and clinical significance are built upon this paradigm.

### 3.2 RANK–RANKL–OPG Axis (NF‐κB/NFATc1 Core)

The major regulating node of osteoclastogenesis and bone resorption is the RANKL–RANK–OPG signalling axis, which operates within a larger, intricately linked signalling network that incorporates hormonal, inflammatory, mechanical, and metabolic inputs in the bone remodelling unit (BMU) [53]. Osteocytes, osteoblasts, stromal cells, and activated immune cells produce RANKL, which binds to its receptor RANK on osteoclast precursors. This causes TRAF6‐dependent activation of the NF‐κB, MAPK and calcium–calcineurin pathways, which in turn triggers NFATc1‐driven transcriptional programs that support osteoclast differentiation and resorptive activity [54, 55]. OPG acts as a protective shield for the skeleton. It neutralises RANKL, the ‘bone‐breaking’ molecule, to maintain healthy, steady bone turnover [56]. This equilibrium is upset in osteoporosis as a result of several risk factors coming together on this signalling axis. While ageing‐related ‘inflammaging’ further increases osteoclastogenesis through persistent elevation of TNF‐α, IL‐1β and IL‐6, oestrogen shortage increases RANKL expression and decreases OPG through NF‐κB–mediated inflammatory signalling [57]. By encouraging osteoblast apoptosis and boosting pro‐resorptive signalling, excess glucocorticoids and oxidative stress also distort the RANKL/OPG ratio [58]. Significantly, there is a lot of interaction between the RANKL pathway and other important signalling systems. For example, inflammatory cytokines inhibit Wnt activity through DKK1 and sclerostin, which reinforces RANKL‐driven bone loss, while Wnt/β‐catenin signalling indirectly suppresses osteoclastogenesis through OPG upregulation [59, 60]. Furthermore, the significance of osteoimmunology in pathological bone resorption is highlighted by immune cell‐derived RANKL, especially from Th17 cells, whereas regulatory T cells provide protection through anti‐inflammatory cytokines. The necessity for integrated, multi‐pathway treatment approaches is highlighted by the fact that, despite its crucial role, therapeutic targeting of this route, as demonstrated by denosumab, displays both success in fracture reduction and limits such rebound bone loss and long‐term safety concerns [61, 62]. Collectively, the RANKL–RANK–OPG axis should be understood not merely as an isolated pathway, but as a dynamic signalling hub where systemic risk factors converge to drive osteoclast‐mediated bone loss in osteoporosis.

### 3.3 Wnt/β‐Catenin Signalling in Osteoporosis

Wnt/β‐catenin signalling integrates mechanical, hormonal, inflammatory and metabolic signals to regulate osteoblast development and indirectly inhibit osteoclastogenesis, serving as a key regulatory hub within the bone remodelling unit [41]. Canonical Wnt ligands trigger signalling by binding to Frizzled (FZD) receptors and LRP5/6 co‐receptors, activating Dishevelled (DVL) and causing the destruction complex (Axin/APC/GSK‐3β) to disassemble or deactivate. This prevents the phosphorylation‐dependent degradation of β‐catenin, allowing it to accumulate, translocate to the nucleus, and activate target genes [63]. In the nucleus, stabilised β‐catenin interacts with TCF/LEF transcription factors to activate the expression of key osteogenic genes, including Runx2 and Osterix (Sp7), which drive mesenchymal stem cell differentiation into osteoblasts and promote subsequent bone formation [64]. Concurrently, Wnt signalling boosts osteoprotegerin (OPG) expression, which acts as a decoy receptor to neutralise RANKL and inhibit osteoclast differentiation, thereby maintaining bone remodelling balance [65]. In osteoporosis, the Wnt/β‐catenin signalling pathway which is critical for bone formation is functionally suppressed. This inhibition is driven by increased levels of endogenous antagonists, primarily sclerostin (encoded by SOST) and Dickkopf‐1 (DKK1). These antagonists bind to LRP5/6 receptors, blocking Wnt signalling and decreasing the activity of β‐catenin, ultimately leading to reduced bone density [66]. Ageing, oestrogen deficiency, and chronic inflammation drive skeletal decline by elevating Wnt inhibitors, which disrupts osteoblastogenesis and impairs bone formation [67]. This loss is largely mediated by oxidative stress, which induces FOXO‐mediated transcriptional programs that divert β‐catenin away from Wnt signalling. Oestrogen deficiency exacerbates this mechanism by increasing oxidative stress and upregulating Wnt inhibitors, further accelerating bone resorption [68]. Pro‐inflammatory cytokines, specifically TNF‐α and IL‐6, drive uncoupled bone remodelling by inhibiting Wnt signalling while simultaneously upregulating RANKL expression to enhance osteoclast activity [69]. Mechanical unloading such as that experienced during immobilisation, bed rest or sedentary behaviour results in the impairment of Wnt activation within osteocytes. This lack of mechanical stimulation leads to an increase in sclerostin (a Wnt antagonist encoded by the SOST gene) levels, which subsequently inhibits bone formation and drives disuse‐induced bone loss [70]. Wnt signalling does not function in isolation; rather, it is a key component of a complex, interconnected regulatory network. It exhibits tight crosstalk with major signalling pathways such as RANKL–RANK–OPG (regulating osteoclastogenesis), parathyroid hormone (PTH), and TGF‐β/BMP to collectively orchestrate skeletal development, bone remodelling, and homeostasis [71]. Targeting Wnt signalling, particularly via sclerostin inhibitors like romosozumab, shows promise in enhancing bone formation and reducing fracture risk. However, potential cardiovascular risks and variable patient responses necessitate careful clinical application. Despite these advancements, significant gaps remain regarding the context‐dependent dynamics and long‐term safety of Wnt‐targeted therapies, necessitating further research into this critical pathway [72].

### 3.4 Parathyroid Hormone (PTH)/PTH1R (cAMP/PKA vs. Sustained Ca^2+^/PKC)

Parathyroid hormone (PTH) acts through the PTH1R receptor to exert a dual, opposite effect on bone remodelling, anabolic (building bone) or catabolic (breaking down bone), with the outcome entirely dependent on the mode and duration of exposure [33, 41]. Intermittent parathyroid hormone (PTH) stimulation preferentially activates the cAMP/protein kinase A (PKA) signalling pathway, fostering osteoblast differentiation, survival and anabolic bone formation. This anabolic signalling cascade increases bone mass by upregulating essential osteogenic genes, such as Runx2 and Osterix. Furthermore, intermittent PTH inhibits osteoblast apoptosis (programmed cell death), prolonging the lifespan of mature osteoblasts and thereby increasing the overall rate of bone formation [73, 74]. Conversely, sustained PTH exposure triggers catabolic pathways by promoting prolonged intracellular Ca^2+^ elevation and activating protein kinase C (PKC) [75]. Prolonged activation of the Ca^2+^/PKC pathway promotes osteoclast formation and bone destruction by upregulating RANKL and reducing osteoprotegerin (OPG). Ultimately, the interplay between these pathways determines the net impact of PTH on bone tissue, favouring either formation or resorption [75, 76]. Recent studies indicate that the temporal dynamics of PTH1R signalling extend beyond the cell surface, involving receptor internalisation and sustained endosomal signalling that modulate downstream effects. Furthermore, crosstalk with the Wnt/β‐catenin signalling pathway strengthens the anabolic response to intermittent PTH, leading to increased osteoblast activity [73]. Conversely, the sustained stimulation of Ca^2+^/PKC signalling interferes with the necessary crosstalk between bone cells, resulting in the reduced bone formation characteristic of osteoporosis. Recognising these differential pathways enabled the development of PTH‐based therapeutics like teriparatide. These agents leverage intermittent administration to favour anabolism over catabolism, reversing skeletal fragility [77].

### 3.5 Sex Steroids: Oestrogen/Androgen Signalling

Oestrogen and androgen signalling are essential for bone homeostasis, and the decline of these hormones is a primary cause of osteoporosis [78]. Oestrogen primarily acts through oestrogen receptors (ERα and ERβ) to suppress osteoclastogenesis by downregulating RANKL and upregulating osteoprotegerin (OPG) in osteoblasts [79]. Oestrogen supports bone formation by enhancing osteoblast survival, specifically by mitigating oxidative stress and apoptosis. Conversely, postmenopausal oestrogen deficiency drives bone resorption by elevating inflammatory cytokines, such as TNF‐α and IL‐6 [80]. The molecular pathways by which ERα signalling exerts these cationic effects include inhibition of NF‐κB and NFATc1, and attenuated osteoclast differentiation and survival through the increased induction of pro‐apoptotic mediators such as Bax and the inhibition of the anti‐apoptotic Bcl‐2 [81, 82]. Androgens support bone health by directly promoting periosteal formation via androgen receptors (AR) and indirectly managing osteoclast‐driven resorption. Additionally, testosterone acts as a precursor that is aromatised into oestrogen, providing crucial bone‐protective effects in both men and women [83]. Recent findings indicate that sex steroids modulate the Wnt/β‐catenin pathway, playing a crucial role in regulating osteoblast differentiation and functional activity. Oestrogen drives Wnt/β‐catenin activity through ERα‐mediated molecular signalling and promotes the stabilisation of β‐catenin and transcription of genes important for osteogenesis, like Runx2 and Osterix. Moreover, there is a small increase in osteoprotegerin (OPG) production that neutralises RANKL and limits osteoclastogenesis [84]. Furthermore, oestrogen is vital for maintaining bone strength by regulating osteocyte viability and mechanotransduction. As oestrogen enhances the mechanosensitivity of osteoblasts and osteocytes through regulation of integrin‐linked kinase (ILK) and focal adhesion kinase (FAK) signalling pathways to optimise their ability to sense and respond to mechanical forces that enhance bone formation. The deficiency of these sex steroids disrupts this integrated signalling network, resulting in accelerated bone turnover and increased skeletal fragility [84].

### 3.6 Glucocorticoid Receptor (GR) Signalling in Osteoporosis: Bone Loss and Cellular Dysfunction

Glucocorticoids regulate bone metabolism through the glucocorticoid receptor (GR), and prolonged exposure is a leading cause of secondary osteoporosis. Upon translocation of GR to the nucleus, where it modulates the transcription of genes that cause quick suppression of bone formation and an increase in bone resorption, which was [85] driven by factors like Bim, PUMA, reactive oxygen species (ROS) and endoplasmic reticulum (ER) stress [36, 86]. This process inhibits osteogenesis and induces apoptosis in osteoblasts and osteocytes, resulting in significantly reduced bone formation [87]. Excess glucocorticoid (GC) signalling severely compromises bone homeostasis by directly suppressing osteoblast‐mediated bone formation and accelerating the apoptosis of osteoblasts and osteocytes, contributing to a ‘frozen’ bone state with reduced remodelling and strength. Molecularly, GCs inhibit key osteogenic pathways, most notably by blocking Wnt/β‐catenin signalling via upregulation of sclerostin and DKK‐1, while simultaneously forcing a differentiation shift from osteoblasts towards adipocytes. The prolonged suppression of osteoblastogenesis, coupled with enhanced osteoclast lifespan, results in significant impairment of bone microarchitecture and increased fracture risk [88]. Simultaneously, glucocorticoids indirectly extend osteoclast survival by altering the RANKL/OPG ratio to favour bone resorption. Another mechanism where the activity of HIF‐1α/2α and the integrity of the angiogenic–osteogenic coupling decrease by GCs. This suppression disrupts the maintenance of type‐H vasculature and decreases the delivery of oxygen and nutrient support to regions of bone formation. As a result, the osteoblast population declines and the mechanical quality of bone matrix is weakened, leading to impaired bone regeneration and an increase in the fragility of the bone [89]. Additionally, GC disrupts the calcium absorption mechanism by acting through duodenal TRPV6 and CaBP‐9ƙ genes, which appear to be major regulators for calcium absorption [90]. Furthermore, glucocorticoid receptor (GR) activation promotes oxidative stress and disrupts mitochondrial function, which further contributes to skeletal fragility [91, 92]. Recent evidence highlights that glucocorticoid receptor (GR) signalling disrupts bone cell homeostasis by interfering with autophagy regulation. Beyond these genomic effects, cytoplasmic GR signalling induces rapid, non‐genomic responses, including altered calcium handling. Collectively, chronic glucocorticoid usage causes significant bone loss and substantially elevates the risk of fractures risk [93].

### 3.7 TGF‐β/BMP–SMAD Signalling

TGF‐β and BMP signalling pathways, acting through SMAD‐dependent transcription, are key regulators of bone remodelling. By modulating osteoblast and osteoclast activity, these pathways control skeletal homeostasis. Dysregulation of this signalling results in various skeletal pathologies [94]. By activating the canonical SMAD2/3 pathway, TGF‐β stimulates the proliferation and early differentiation of osteoprogenitors while inhibiting terminal differentiation, mineralisation and osteocyte transition, thus contributing to compromised bone formation under disease conditions [95]. While TGF‐β has distinct roles, BMP signalling mediated by SMAD1/5/8 promotes osteoblast differentiation and bone matrix mineralisation. The breakdown of this TGF‐β/BMP balance is now recognised as a key factor in osteoporosis, especially during ageing and oestrogen‐deficient states [96]. High levels of TGF‐β activity accelerate osteoclast recruitment and disrupt bone remodelling, as the increased release of TGF‐β during resorption drives a feedback loop that undermines the coupling of bone formation and breakdown [97]. The interaction of TGF‐β/BMP signalling with Wnt/β‐catenin and RANKL–OPG pathways creates a comprehensive network that regulates bone homeostasis. Recent studies underline the influence of epigenetic regulation and SMAD‐independent signalling on skeletal pathway outcomes [85]. Although targeting this network holds therapeutic potential, significant challenges regarding specificity and safety persist.

### 3.8 Notch–Hedgehog Signalling

The Notch and Hedgehog signalling pathways play a key role in skeletal growth and bone health by guiding cell decisions. Notch signalling keeps bone marrow progenitors in an undifferentiated, proliferative state, maintaining a pool for future bone formation by suppressing Runx2‐mediated osteoblast differentiation [98]. Within osteoprogenitor cells, Notch signalling activation functions to maintain osteoprogenitor and/or pre‐osteoblastic states by preventing terminal osteoblastic differentiation. Mechanistically, this inhibitory action occurs through upregulating transcriptional repressors HEY and HES that negatively regulate osteogenic transcription factors like Runx2 and Osterix, thus limiting late‐stage osteoblastic maturation [99, 100]. While aberrant Notch activation inhibits osteoblast maturation and promotes indirect osteoclastogenesis via altered RANKL expression, Indian hedgehog (Ihh) signalling drives osteoblast lineage commitment and endochondral ossification [101]. A major function of Ihh is to create a feedback loop with parathyroid hormone‐related protein (PTHrP) in order to maintain an appropriate balance between chondrocyte proliferation and differentiation to hypertrophy. Ihh signalling promotes the maintenance of a pool of proliferating chondrocytes and induces expression of the transcription factor Runx2, which drives osteoblast differentiation within the perichondrium, linking chondrogenesis and osteogenesis [102]. Consequently, disruption of this Hh pathway impairs osteoblast differentiation, contributing to reduced bone formation in osteoporotic conditions [103, 104]. Emerging evidence indicates that Notch and Hedgehog (Hh) pathways engage in significant crosstalk, with Notch often antagonising Hh‐driven osteogenesis under pathological conditions. These pathways, alongside Wnt/β‐catenin and TGF‐β, construct a complex regulatory network that manages bone remodelling. Factors like ageing, chronic inflammation and hormonal shifts disrupt this Notch–Hh equilibrium, driving the process towards accelerated bone loss [105]. While targeting this crosstalk presents a promising therapeutic avenue, the context‐specific nature of these signalling mechanisms and potential safety issues create substantial translational hurdles.

### 3.9 PI3K–Akt–mTOR and Autophagy

As a central regulator of bone homeostasis, the PI3K‐Akt–mTOR axis governs osteoblast survival and proliferation by integrating nutrient and growth factor signalling [50]. While PI3K‐Akt signalling induces osteoblast differentiation and anabolic bone formation through mTORC1, this same mechanism can be detrimental if overactive. Hyperactivation of mTOR suppresses necessary autophagy, disrupting cellular homeostasis and impairing osteocyte/osteoblast survival under stress conditions [106]. Defective autophagy causes osteoporosis by triggering oxidative stress and reducing mineralisation capacity due to organelle accumulation. In contrast, PI3K‐Akt signalling exerts distinct effects within the bone unit, enhancing survival and resorptive activity specifically in osteoclasts, highlighting the dual, cell‐type‐dependent role of autophagy‐related pathways in bone metabolism [106, 107]. Ageing, glucocorticoid exposure, and oestrogen deficiency dysregulate PI3K‐Akt–mTOR signalling, shifting the balance towards reduced autophagy and increased bone loss. Importantly, crosstalk exists between this pathway and Wnt/β‐catenin and AMPK signalling, highlighting a broader metabolic network controlling bone homeostasis [108]. While targeting mTOR and autophagy via agents like rapamycin or AMPK activators shows therapeutic potential, the dual effects of these pathways on bone cells necessitate precise dose and context optimisation.

### 3.10 Oxidative Stress, Inflammation and Epigenetic Regulation

Oxidative stress, acting through inflammatory and epigenetic pathways, drives osteoporosis by disrupting bone homeostasis. High levels of ROS, in particular, inhibit osteogenesis via suppressed Runx2 and activated FoxO signalling while concurrently driving osteoclastogenesis, resulting in excessive bone resorption [109]. Chronic inflammation, driven by pro‐inflammatory cytokines like TNF‐α, IL‐1β and IL‐6, accelerates bone resorption by stimulating RANKL expression and activating osteoclasts (Table 3). This process is amplified by a feed‐forward loop where oxidative stress and immune signalling converge on the NF‐κB pathway, rapidly driving pathologic bone loss. Epigenetic mechanisms, including DNA methylation, histone modifications, and non‐coding RNAs (miRNAs, lncRNAs), regulate bone metabolism by controlling osteoblast and osteoclast gene expression without changing the DNA sequence. These processes modulate chromatin structure, impacting bone formation, resorption, and regeneration to maintain skeletal homeostasis [110]. For example, dysregulated miRNAs (e.g., miR‐21, miR‐29) modulate Wnt and RANKL signalling, directly affecting bone formation and resorption balance. Ageing, oestrogen deficiency, and environmental stressors drive epigenetic changes that amplify inflammation and oxidative stress, thereby accelerating the progression of osteoporosis [111]. These processes represent a systems‐level network, interacting with PI3K–Akt, Wnt/β‐catenin and TGF‐β pathways. Targeting inflammation, oxidative stress and epigenetic modifiers is promising, but hampered by challenges in specificity and long‐term safety [111, 112].

**TABLE 3:** Relative Risk of developing osteoporosis based on ethnicity.

Ethnicity/race	Relative risk
Black people	0.46 (95% CI, 0.43–0.48, *p* < 0.0001)
Hispanics	0.66 (95% CI, 0.55–0.79, *p* < 0.0001)
Asian American	0.55 (95% CI, 0.45–0.66, *p* < 0.0001)
American Indians	0.80 (95% CI, 0.41–1.58, *p* = 0.3436)

### 3.11 Hypoxia/HIF–VEGF Axis (Osteogenesis–Angiogenesis Coupling)

The Hypoxia‐inducible factor (HIF) vascular endothelial growth factor (VEGF) axis acts as a critical regulator that couples angiogenesis (blood vessel formation) with osteogenesis (bone formation) during bone remodelling. When bone tissues experience reduced oxygen (hypoxia), osteoblasts and osteocytes stabilise HIF‐1α, which in turn induces the expression of VEGF. HIF‐1α and HIF‐2α will initiate glycolytic enzyme expression to shift cellular metabolism towards aerobic glycolysis, which meets the high‐energy demands of osteoblast precursors. HIF signalling also induces the formation of specialised type‐H capillaries, a specialised subtype of endothelial cells that are distinguished by their high expression of CD31 and Endomucin (Emcn) as an endothelial marker [113, 114]. This process promotes vascular invasion, ensuring the necessary nutrient supply and bringing osteoprogenitor cells to the site, ultimately driving bone formation [114]. VEGF not only drives angiogenesis but also directly enhances osteoblast differentiation and survival, highlighting its dual role in skeletal homeostasis [115]. Bone loss in osteoporosis often stems from broken communication between blood vessels and bone cells, specifically due to weakened HIF‐VEGF signalling. Factors such as ageing, low oestrogen, and steroids reduce HIF‐1α activity, hindering the blood vessel growth needed to form new bone. This mechanism is complex, involving interactions with other signalling pathways (Wnt/β‐catenin, PI3K‐Akt, and Notch) that manage bone metabolism and structure [113]. Furthermore, hypoxia‐mediated signalling disrupts bone remodelling balance by indirectly driving osteoclast function via vascular and inflammatory mediators. While therapeutic targeting of the HIF–VEGF axis can improve vascularization and bone formation, this approach requires precise modulation to prevent the risks associated with aberrant angiogenesis [116].

## 4 Risk Factors Driven Mechanism

There are some factors can be altered to reduce the risk of osteoporosis and few other have no contol. The factors that can be modified to reduce the risk are inadequate nutrition, lack of physical activity, weight loss, cigarette smoking, alcohol consumption, air pollution, gut health, and stress, and unmodifiable risk includes age, gender, ethinicity, and cormorbidies.

### 4.1 Vitamin D

**FIGURE 5:**
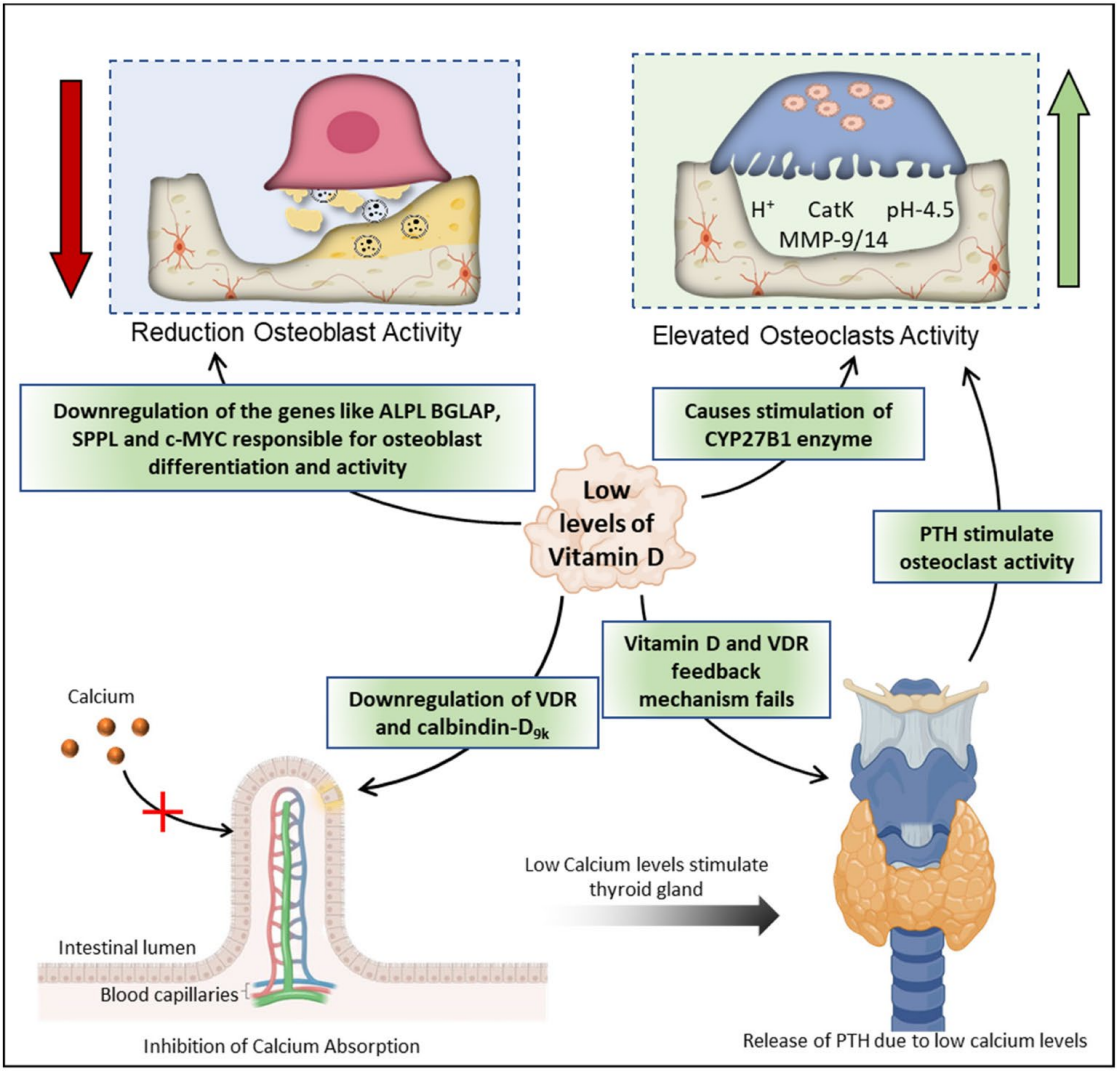
Impact of vitamin D deficiency on osteoporosis development.

Vitamin D has a key role in the absorption of calcium from the intestine and a deficiency of vitamin D will cause osteoporosis (Figure 5). The enterocytes contain certain receptors like Vitamin D receptor (VDR) and localised proteins like calcium‐binding protein calbindin‐D_9k_, responsible for calcium absorption, and it is regulated by Vitamin D. It also regulates tight junction proteins of the claudin family and facilitates the paracellular transport of calcium through improved junction ion permeability [117, 118]. It was reported that Vitamin D has a positive effect on bone formation through 1,25D3‐induced c‐MYC expression. It is associated with the stimulation and release of VEGF and blood vessel formation that will support the differentiation of the osteoblast cells and bone formation. It was also observed that the VDR on osteoblast cells was responsible for the modification of the gene expression of alkaline phosphatase (ALPL), osteocalcin (BGLAP) and osteopontin (SPPL), which are key components for the formation of the bone matrix [119]. Controversial, the finding by Zarei et al. mentioned that vitamin D has decreased the size and number of the osteoclast, but the VDR and the vitamin D metabolite, 1,25(OH)_2_D_3_ have the opposite effect. The enzyme, CYP27B1, expressed by osteoclasts, metabolises the Vitamin D into 1,25(OH)_2_D_3_, which stimulates osteoclastogenesis. They also found that there was a downregulation of NFATC1 and TM7SF4 expression which are the key markers of the osteoclast fusion. However, the increased bone resorption activity observed was due to the formation of smaller osteoclasts over a few larger multinucleated osteoclasts [120]. There was also reported literature on the relation between lower Vitamin D, Parathyroid hormone (PTH), and osteoporosis development. The VDR present on the parathyroid gland acts as a negative feedback mechanism. During the drop in calcium levels, PTH is released to stimulate the CYP27B1 enzyme. But, the vitamin D liganded with VDR to inhibit the release of PTH and simultaneously increase the intestinal calcium absorption [121]. Recently, Xiu‐quan Qu and their research group found that insufficient intake of Vitamin D and calcium will lead to parathyroid hyperplasia which reflects on the increase in the PTH secretion and development of the osteopoeosis [122].

### 4.2 Physical Activity

Exercise has been demonstrated to attenuate the progression of osteoporosis by modulating bone cell function through multiple mechanotransductive pathways. Osteocytes are capable of directly sensing and responding to mechanical stimuli through a variety of specialised mechanosensors. These include components of the Wnt signalling, integrins that detect extracellular matrix stress, connexins that form intercellular gap junctions facilitating mechanosignal propagation, and purinergic receptors that mediate ATP‐dependent signalling [123, 124, 125, 126]. Lack of regular physical activity leads to a progressive decline in muscle mass and strength, which in turn compromises joint stability and contributes to bone fragility [127, 128]. Along with signalling pathways, anabolic hormones (GH, IGF‐1, oestrogen and PTH) and osteogenic intermittent stress are amplified in response to mechanical stress or exercise. These stimulations were produced as a physical stress adaptive mechanism to the bone to increase size, shape and bone density. So, lack of physical activity will diminish these adaptive anabolic mechanisms and may increase the risk of osteoporosis [129].

### 4.3 Smoking and Alcohol Consumption

Along with lack of physical activity, Smoking and alcohol consumption are lifestyle‐based modifiable risks. Smoking has direct and indirect negative effect on the bone structure and formation. They disturb the normal physiology associated with calcium absorption, 25‐hydroxy‐vitamin D, parathyroid hormone and RANK‐RANKL‐OPG responsible for osteogenesis. Alongside, alcohol consumption shows an undesirable effect on bone mineral density. It affects the bone growth, and homeostasis of calcium and magnesium via excessive excretion and reduces the 25‐hydroxyvitamin D_3_ and 1,25‐dihydroxyvitamin D_3_ serum levels. Comprehensively, smoking and excessive alcohol intake increase the risk of osteoporosis [130, 131]. Marinucci et al. have investigated the impact of nicotine on osteoblasts, where they found that the release and accumulation of an advanced glycation end product called Hydromidazolone (MG‐H1). MG‐HI act as a pro‐apoptotic agent; higher levels of release and accumulation cause H_2_O_2_ overproduction and Transglutaminase 2 (TG2) downregulation‐dependent NF‐ƙB desensitisation, triggering the mitochondrial apoptotic pathway, leading to osteoblast cell death [132]. The meta‐analysis by Law et al. has found that there is a comparatively higher risk of hip fracture, 37% for smokers to 22% non‐smokers at the age of 90 [133]. Contrarily, a recent cross‐sectional study in the Persian population found no significant correlation between smoking and alcohol consumption [134]. A similar observation was reported by Thompson et al., considering the U.S. population, where there is no significant relevance with men, whereas the prevalence was twice in the women population due to smoking [135]. However, apart from the negative influence of smoking on the osteoblast reported, Qui et al. has explored the impact of smoking on the osteoclast as well. The TRAP staining shows a high number of multinucleated areas for the cigarette extract‐treated group compared to the control group. Further investigation in the studies shows that the cigarette has no influence on the osteoclast differentiation and proliferation, but it prolongs the survival period by inhibiting apoptosis through release of cytochrome C and caspase 3 which markedly decrease the mtROS [136].

### 4.4 Mental Stress

Stress is also a modifiable risk factor for the cause of osteoporosis, and it acts as the stimulus for various adaptive mechanisms. The hypothalamus of the brain is involved in bone remodulation in response to stress. It controls and releases certain neurotransmitters and peptides like adrenaline, noradrenaline, neuropeptide Y, and neuromedin U, which are involved in osteoblast proliferation and bone remodelling. The initial stress possibly has a positive effect on bone formation, but chronic exposure to the stress leads to a negative effect on bone remodelling and induces osteoporosis [137]. Also, psychological stress causes the dysregulation of the hypothalamus‐pituitary–adrenal axis, which leads to hypersecretion of cortisol. The interaction of cortisol with glucocorticoid receptors stimulates glucocorticoid‐induced osteoclast stimulation and osteoblast inhibition. Through the upregulation of RANKL and downregulation of OPG, osteoclast differentiation and activity have been enhanced and, by inducing apoptosis in the osteoblasts, bone formation has been downregulated. The neuropeptide Y and neuromedin U are the neurotransmitters released in response to manage stress, but due to their negative effect on bone formation, osteoporosis will develop [138].

### 4.5 Age

For the first three decades of life, the balance will be maintained between bone formation and resorption. Later there will be a drop in the levels of sex hormones (oestrogen and testosterone) and age‐related reduction of intestinal calcium absorption capability, elevation in the bone resorption process, and development of osteoporosis. the reduction of calcium absorption causes higher levels of parathyroid hormones in the circulation and further increases osteoclast activity and bone resorption [139]. Premature menopause and the use of medications that suppress gonadal function—such as aromatase inhibitors and gonadotropin‐releasing hormone (GnRH) analogs—are associated with reduced BMD and an increased risk of fragility fractures [140]. Ageing and postmenopausal condition cause the drop in oestrogen levels, that can increase reactive oxygen species (ROS), which causes β‐catenin to switch from partnering with TCF to teaming up with FOXO [141]. This shift favours survival over cell proliferation and leads to a decrease in osteoblast formation and function. The Nrf2 antioxidant pathways struggle to keep up, and as mitochondrial function declines and DNA gets damaged, p53/p21 and the senescence‐associated secretory phenotype (SASP) are activated. This process encourages the formation of osteoclasts while hindering the activity of osteoblasts [142, 143, 144].

### 4.6 Gender

In comparison with males and females, females are more susceptible to osteoporosis. The various meta‐analyses indicate that women are more susceptible to develop it because men have greater bone size, a drop in oestrogen levels after menopause, and also the increased compensation of the periosteal bone formation upon endosteal bone loss in men [139, 145, 146]. One among the sex hormones, androgens, exert dual effects on bone metabolism directly through activation of the androgen receptor (AR) and indirectly via aromatisation to oestrogens that signal through oestrogen receptor‐α (ERα) [147]. And at the cellular level, testosterone and 5α‐dihydrotestosterone (5α‐DHT) regulate osteoblast gene expression and suppress osteoclast resorptive activity [148]. Moreover, sex hormones modulate the secretion of cytokines and growth factors by skeletal cells, including macrophage colony‐stimulating factor, interleukin‐1, interleukin‐6, tumour necrosis factor‐α, RANKL and osteoprotegerin. These mediators collectively govern the balance between bone formation and resorption, underpinning the critical role of androgens in maintaining skeletal homeostasis [149].

### 4.7 Gut Health

The cohort study conducted and published in 2023 by Hansen et al. has proven that gut health has a significant impact on bone fractures and osteoporosis. Celiac disease (CD) is an autoimmune disease triggered by the ingestion of gluten. They found that the population having CD is 5.39 times more likely to develop osteoporosis [150]. The gut‐microbiota also has influence on the development of osteoporosis. Microbiota‐derived short‐chain fatty acids (SCFAs) play a significant role in bone health by influencing immune responses (lowering TNF‐α and IL‐6), sending endocrine signals (boosting IGF‐1), and possibly acting directly on osteoblasts and osteoclasts through GPR41 and GPR43. When there is an imbalance in gut bacteria due to factors like diet, antibiotics, or ageing, it can lead to increased inflammation and a decrease in the efficiency of calcium and Vitamin D absorption and metabolism, which may increase the risk of osteoporosis [151, 152, 153]. The mechanism involved in the development of osteoporosis through gut health is osteoclastogenesis. Upon disturbance of the immune system and altered CD4+ T cell portions, the upregulation of cytokine levels specifically receptor activator of nuclear factor kappa‐B ligand (RANKL) expression occurs. These elevated levels induce the differentiation of the osteoclast cells and their activity, leading to the development of osteoporosis [154].

### 4.8 Medicines and Comorbidities

Certain therapeutic interventions and comorbidities were responsible for the development of osteoporosis. Glucocorticoid is a hormone produced and released by the adrenal cortex upon the stimulation of the suprachiasmatic nerve in the hypothalamus. It has a role in various physiological functions, and bone formation and remodelling is one among them. The endogenous glucocorticoid has a positive effect on bone formation, whereas, in long‐term exposure, it has a negative effect on bone remodelling. Instead of osteoblast formation, glucocorticoids influence the pluripotent precursor cells to convert into adipocytes. The deviation in cell formation was mediated by the glucocorticoid upregulated pathways like PPARγ2, KLF15, C/EBPα and aP2. On the other hand, osteoclast formation is enhanced upon the upregulation of the RANKL pathway and other factors like IL‐6 and interferon‐β. It has a negative effect on calcium homeostasis through an increase in the renal excretion of calcium, and it downregulates the calcium transporter expression in the duodenal region, which results in the reduction of calcium absorption [155, 156, 157, 158]. The Parathyroid hormone (PTH) is secreted by the parathyroid gland, which affects bone remodelling. This hormone secretion was stimulated by low levels of serum calcium, which leads to the release of calcium from the bone matrix. Contradictorily, it shows both catabolic effects on cortical bone and anabolic effects on cancellous bone. However, in the case of hyperparathyroidism, the elevated parathyroid hormone levels cause osteoporosis [159, 160, 161, 162]. The crosstalk between PTH and Wnt signalling, involving LRP6 phosphorylation and Dishevelled (Dvl) stabilisation, contributes to its dual actions, while PTH‐induced shifts in the OPG/RANKL balance further dictate whether bone formation or resorption predominates [163]. Clinical evidence indicates that suppressed serum Thyroid stimulating hormone (TSH) levels and a history of hyperthyroidism are strongly associated with an elevated risk of hip and vertebral fractures. Experimental findings suggest that TSH itself directly inhibits bone resorption, implying that TSH suppression by excess thyroid hormones may contribute to bone loss [149, 164]. Other diseases like gastrointestinal disease, autoimmune diseases, liver disease and kidney disease, or some of the medications used in the treatment of these diseases, can cause osteoporosis [165, 166, 167, 168].

### 4.9 Ethnicity

Around the world, black women have a lower risk of osteoporosis. Even though the serum levels and intake of calcium and vitamin D are lower than those of American, White and Asian women, black women are less likely to develop osteoporosis. The possible reason for this contradictory phenomenon may be genetics, but the actual reason or mechanism is not well understood [169, 170]. The observation reported in 2023 considered the U.S. population to find the correlation with osteoporosis and smoking. It was found that there is a correlation with smoking and osteoporosis; interestingly, they found the correlation with gender and ethnicity as well. As most reports have shown, women are more prone to osteoporosis, which was the same outcome from their study, and there was no association of smoking and osteoporosis in men, except non‐Hispanic Black men, which was contradictory to the few previous reports in view of ethnicity‐associated osteoporosis risk [135]. Another meta‐analysis by Yueyang Bao et al. shows the different relative risk of osteoporosis in different ethnicities mentioned in Table 3, indicating that white people have a high risk of developing osteoporosis in comparison with other races and ethnic groups [171]. The possible variation in ethnicity is possibly due to genetics. Aldkdlkd et al. have done mapping and found out the single nucleotide variations (SNVs) that possibly have a greater impact on BMD. They found around 125 SNVs affect the femoral neck BMD, 99 SNVs for the spine BMD and 83 SNVs for hip BMD. Among them, RNA gene LINC02370, ZNF family genes and ZDHHC family genes have a positive association with BMD, ZNF family genes across skeletal sites. Targeting these genes can lead to the development of newer therapeutic options for the management of osteoporosis [172].

## 5 Conclusion

Osteoporosis is one of the most significant bone disorders affecting people around the world due to the weakened bone structure and lower bone mineral density, which makes individuals more susceptible to fractures. This detailed review sheds light on the intricate relationship between the various macro and micro‐pathophysiological factors that contribute to the onset and progression of osteoporosis. The condition arises from a fundamental imbalance in bone remodelling, where the activity of osteoclasts, which break down bone, outpaces the work of osteoblasts and osteocytes, leading to a weakened skeletal framework. At the macroscopic level, several critical pathways maintain bone health. The RANK‐RANKL‐OPG axis serves as a central regulatory mechanism, where dysregulation leads to enhanced osteoclastogenesis and accelerated bone resorption. Hormonal influences, particularly oestrogen and testosterone, play pivotal roles in maintaining the delicate balance between bone formation and resorption, with their deficiency being a primary driver of postmenopausal and age‐related osteoporosis. The transcription factor Runx2 emerges as another crucial regulator, controlling both Wnt signalling pathways and OPG expression, thereby influencing overall bone remodelling dynamics. The micro‐pathophysiology reveals an intricate network of cellular and molecular mechanisms. The bone remodelling unit (BMU) concept demonstrates how osteoclasts, osteoblasts, and osteocytes coordinate within basic multicellular units to maintain skeletal homeostasis. Disruption of this coupling mechanism, whether through increased osteoclast activity, decreased osteoblast function, or compromised osteocyte mechanosensing, contributes significantly to osteoporotic bone loss. Key signalling pathways including Wnt/β‐catenin, PTH/PTH1R, TGF‐β/BMP‐SMAD and PI3K‐Akt–mTOR pathways collectively regulate bone cell fate determination, differentiation, and survival. The multifactorial nature of osteoporosis is evident through the identification of modifiable and non‐modifiable risk factors. While age, gender, and genetic predisposition represent unchangeable risks, lifestyle modifications including adequate nutrition, regular physical activity, smoking cessation, and alcohol moderation offer significant preventive potential. Secondary causes, particularly glucocorticoid exposure and hyperparathyroidism, highlight the importance of considering iatrogenic and comorbid contributions to bone loss. Contemporary understanding emphasises the role of systemic factors in osteoporosis pathogenesis. Oxidative stress, chronic inflammation, and cellular senescence create a microenvironment conducive to bone loss through multiple mechanisms including enhanced osteoclastogenesis, impaired osteoblast function, and compromised bone matrix quality. The emerging gut–bone axis represents a paradigm shift in understanding how microbiota‐derived metabolites influence bone health through immune modulation and endocrine signalling. Additionally, epigenetic modifications and circadian rhythm disruptions add further complexity to the osteoporotic phenotype.

## 6 Future Perspectives

While there has been remarkable progress in understanding the macro‐ and micro‐pathophysiology of osteoporosis, there is an urgent need to translate this understanding of molecular pathology into effective therapeutics that are targeted and patient‐specific. A focus of future research should be on integrative approaches which employ molecular biology, nanotechnology, and drug candidate medicine to tackle both the structural and cellular instabilities of osteoporosis. On the molecular scale, mapping the crosstalk between major pathways such as RANK–RANKL–OPG, Wnt/β‐catenin, PI3K–Akt–mTOR and oxidative stress signalling mechanisms will be critical. Multi‐omics strategies (transcriptomics, proteomics, metabolomics and epigenomics) will allow for defining patient‐specific signalling signatures and establishing novel therapeutic targets specifically for postmenopausal and glucocorticoid‐induced osteoporosis. Adding miRNA, circRNA and epigenetic modulators to therapeutic paradigms can provide a fine‐tuned approach to regulation of bone remodelling genes and the possibility to leverage precision epigenetic therapies. The field of regenerative and therapeutic medicine is beginning to take great strides in the next decade in the use of bioengineered scaffolds, exosome‐based carriers, peptide‐ or aptamer‐functionalized nanoparticles for the site‐specific delivery of osteoanabolic agents and RNA therapeutics. These advance delivery system hold the potential to improve the bioavailability of the drugs, limit systemic toxicity and promote localised osteogenesis through targeted modulation of both osteoblasts and osteoclasts. Additionally, 3D bioprinting, hydrogel‐based microenvironments, and cell‐laden constructs are promising avenues for restoring bone microarchitecture in high‐grade osteoporotic defects. At a systemic level, understanding the cross‐talk among the gut–bone axis, immune signalling, and circadian regulation provides new interventions for a holistic management of osteoporosis. Modulating gut microbiota using probiotics, prebiotics or postbiotics may add to traditional pharmacologic treatment by reducing inflammation and optimising calcium–vitamin D metabolism. Furthermore, understanding that circadian rhythmicity genes (i.e., BMAL1, CLOCK) also regulate bone turnover may lead to chronotherpeutic strategies to optimise interventions based on the timing of predictable physiologic cycles of bone remodelling. Overall, the future of osteoporosis research and therapy lies in multidimensional molecular mechanisms, advanced biomaterials and digital technologies to move from reactive fracture management towards proactive bone health restoration and preservation.

## Author Contributions

**Prashant Kesharwani:** conceptualization, writing – review and editing, supervision. **Pramoda G.:** writing – original draft. **Kamini Shivhare:** writing – original draft. **Piyush Kumar Gupta:** writing – original draft. **Rahul Shukla:** conceptualization, writing – review and editing, supervision. **Amirhossein Sahebkar:** writing – review and editing.

## Funding

The authors have nothing to report.

## Ethics Statement

The authors have nothing to report.

## Conflicts of Interest

The authors declare no conflicts of interest.

## Data Availability

The authors have nothing to report.
